# The Decay of Disease Association with Declining Linkage Disequilibrium: A Fine Mapping Theorem

**DOI:** 10.3389/fgene.2016.00217

**Published:** 2016-12-12

**Authors:** Mehdi Maadooliat, Naveen K. Bansal, Jiblal Upadhya, Manzur R. Farazi, Xiang Li, Max M. He, Scott J. Hebbring, Zhan Ye, Steven J. Schrodi

**Affiliations:** ^1^Department of Mathematics, Statistics and Computer Science, Marquette UniversityMilwaukee, WI, USA; ^2^Center for Human Genetics, Marshfield Clinic Research FoundationMarshfield, WI, USA; ^3^Biomedical Informatics Research Center, Marshfield Clinic Research FoundationMarshfield, WI, USA; ^4^Computation and Informatics in Biology and Medicine, University of Wisconsin-MadisonMadison, WI, USA

**Keywords:** fine-mapping, linkage disequilibrium, statistical genetics/genomics, two-site model, disease genetics, theoretical genetics, disease association, mode of inheritance

## Abstract

Several important and fundamental aspects of disease genetics models have yet to be described. One such property is the relationship of disease association statistics at a marker site closely linked to a disease causing site. A complete description of this two-locus system is of particular importance to experimental efforts to fine map association signals for complex diseases. Here, we present a simple relationship between disease association statistics and the decline of linkage disequilibrium from a causal site. Specifically, the ratio of Chi-square disease association statistics at a marker site and causal site is equivalent to the standard measure of pairwise linkage disequilibrium, *r*^2^. A complete derivation of this relationship from a general disease model is shown. Quite interestingly, this relationship holds across all modes of inheritance. Extensive Monte Carlo simulations using a disease genetics model applied to chromosomes subjected to a standard model of recombination are employed to better understand the variation around this fine mapping theorem due to sampling effects. We also use this relationship to provide a framework for estimating properties of a non-interrogated causal site using data at closely linked markers. Lastly, we apply this way of examining association data from high-density genotyping in a large, publicly-available data set investigating extreme BMI. We anticipate that understanding the patterns of disease association decay with declining linkage disequilibrium from a causal site will enable more powerful fine mapping methods and provide new avenues for identifying causal sites/genes from fine-mapping studies.

## Introduction

Genetic markers closely linked to disease-causing sites will exhibit association with disease through linkage disequilibrium (Lai et al., 1994; Weiss and Clark, 2002; Morton, 2005; Slatkin, 2008). This is the central idea behind population-based association mapping of disease genes using high density SNP arrays (McVean et al., 2005; Balding, 2006). However, the decay of disease association with declining linkage disequilibrium from a disease-predisposing, functional site has not yet been completely described even though this is a fundamental property of disease genetics. Doing so will provide much needed information concerning the properties of disease genetics and greatly aid experimental designs and statistical methods for identifying functional variants in regions that exhibit disease association.

Although many have argued that genome-wide association studies have been largely unsuccessful in that they have not revealed a large proportion of the heritability from most complex diseases (Latham, 2011), it is certainly clear that numerous loci with impressive statistical evidence for correlation with a wide variety of complex diseases have been identified and replicated (Welter et al., 2014). In a number of instances, these results have provided much needed insight into the biochemical pathways and cellular mechanisms responsible for increasing disease risk (Klein et al., 2005; Cargill et al., 2007; Xavier et al., 2008; Visscher et al., 2012). However, the functional variants underlying the majority of these disease-associated regions have yet to be identified and described (McClellan and King, 2010). The dearth of information concerning functional variants obviously presents a sizable impediment to further dissection of complex disease etiologies and subsequent utility in impacting clinical practice. If genetic and statistical methods can aid in generating either supporting or opposing evidence for the role of functional motifs within a region of disease association, then the progression of human genetics studies can be made much more efficient and potent.

When designing fine mapping genotyping experiments, it is important to select genetic variants and subregions such that the design is well-powered to discover functional variants under two important types of disease models: The first class of model that should be covered by such efforts encompasses models of a causal variant driving a portion, or perhaps all of the disease association within a region. Under this model, varying levels of association signal at different sites are explained by different levels of linkage disequilibrium with causal variants. Hence, given allele frequencies and linkage disequilibrium patterns, one can, in principle, back-calculate the properties of putative functional variants that could be driving an initially observed disease association within the region of interest. Known variants, including those that were not initially interrogated, fulfilling these calculated allele frequency and linkage disequilibrium properties with the initial markers should then be included in a fine-mapping panel. The second model that should be covered by a fine-mapping panel of markers is one of allelic heterogeneity at a functional motif (e.g., a gene) that was originally found to exhibit a disease association signal. Empirical data tends to strongly favor this type of model over an individual variant serving as the sole driving allele within a region (Raychaudhuri et al., 2011; Rivas et al., 2011; Nelson et al., 2012; Kim-Howard et al., 2013; Seddon et al., 2013). Indeed, it is quite typical for studies aiming to fine map regions harboring a GWAS-significant SNP to reveal multiple disease-correlated variants within the same gene. This is not terribly surprising as the site frequency spectrum is expected to contain vast numbers of rare variants in outbred populations, which is accentuated in rapidly expanding demographics (Wright, 1931; Coventry et al., 2010; Keinan and Clark, 2012). Even if there is a small likelihood of any one of these rare variants to exhibit pathogenic effects, the sheer number of variants segregating at a gene trends to produce multiple functional alleles in a sizable population. To cover this class of disease models, one would want to reliably identify the functional motifs tagged by an initial association signal and proceed by exhaustively interrogating variants within those functional motifs. Ultimately, *in vitro* or *in vivo* functional studies will serve to confirm that specific, very rare variants have pathogenic effects. In practice, this two-model approach guiding fine mapping was successfully employed to identify alleles segregating at the *TRAF1-C5* region conferring susceptibility to rheumatoid arthritis (Schrodi et al., 2007a; Chang et al., 2008) and to fine map the *IL23R* region in psoriasis (Garcia et al., 2008).

Here, building upon previous work (Kruglyak, 1999; Pritchard and Przeworski, 2001; Zaykin et al., 2006; Schrodi et al., 2007b, 2009), we prove a simple, analytic relationship between case/control association statistics at two closely-linked sites and the linkage disequilibrium between the two sites under a generalized disease genetics model. The result holds treating the parameters as being fixed. Interestingly, the result is invariant with mode of inheritance parameters. Further, we posit that concurrently considering the patterns of disease-association and the genetic architecture within a region of interest may strengthen the ability to assess the likelihood that a particular variant is indeed causal with regard to inflating the risk of disease. By doing so, one may be better able to prioritize variants for functional follow-up studies. For finite sample sizes, dispersion around this relationship is expected if the parameters are replaced with random variables and we therefore explore this variation in the result through the use of a Monte Carlo simulation. Lastly, we investigate these patterns in experimental data around the *FTO* locus in a large GWAS of extreme BMI.

## Results

### Approximation

Several groups have described the relationship of statistical power at a marker site in linkage disequilibrium with a causal site. In 1999, using the coalescent process to investigate the density of markers necessary for adequate coverage across the genome to detect disease-associated regions, Kruglyak presented the outline of an argument that the sample size necessary to detect association at a marker locus in linkage disequilibrium with a causal site is approximately *S*/*d*^2^, where *S* is the number of samples required to detect disease association at the causal site with a given level of power and *d*^2^ = [*q*(1 − *q*)*p*^−1^(1 − *p*)^−1^]*r*^2^, such that *r*^2^ is the standard measure of linkage disequilibrium between the causal site and the marker site and *q* and *p* are the allele frequencies at the marker and causal sites, respectively (Kruglyak, 1999). Later, Pritchard and Pzreworski performed a derivation showing a similar result, also with regard to power (Pritchard and Przeworski, 2001). Under the Pritchard- Przeworski derivation, the power to detect disease association at a causal site and marker site were found to be approximately the same if the sample size at a marker site is increased by a factor of (*r*^2^)^−1^ over that used in interrogating the causal site. While certainly an intriguing relationship between sample sizes, as it is, the finding may not always have utility in fine mapping applications as most association studies use the same number samples at all sites interrogated. That said, this relationship can be used to motivate related and illuminating properties regarding how fast the disease association signal can be expected to decay as a function of declining linkage disequilibrium from a causal site. Equating the power at the disease-predisposing site to that at the marker site, it follows that,

(1) $$\begin{array}{l} \Phi \left(Z_{D}\sqrt{r^{2}}-Z_{1-\alpha /2}\right)\approx \Phi \left(Z_{M}-Z_{1-\alpha /2}\right); \end{array}$$

where *Z*_*D*_ and *Z*_*M*_ are the normally-distributed *Z*-scores for testing disease-association at the causal site and marker site, respectively; and α is the significance level. Taking the inverse functions and squaring yields the provocative approximation,

(2) $$\begin{array}{l} \chi_{M}^{2}\approx r^{2}\chi_{D}^{2}; \end{array}$$

where $\chi_{D}^{2}$ and $\chi_{M}^{2}$ are the Chi-Square statistics for disease association at the disease and marker sites, respectively. An interesting parallel was described by Luo, Thompson, and Wooliams in the context of marker-assisted selection of quantitative traits where the authors showed that the proportion of the additive variance of a trait due to a marker in linkage disequilibrium with a causal quantitative trait locus, $\sigma_{M}^{2}/\sigma_{A}^{2}$, is equal to *r*^2^ (Luo et al., 1997).

**Figure 1:**
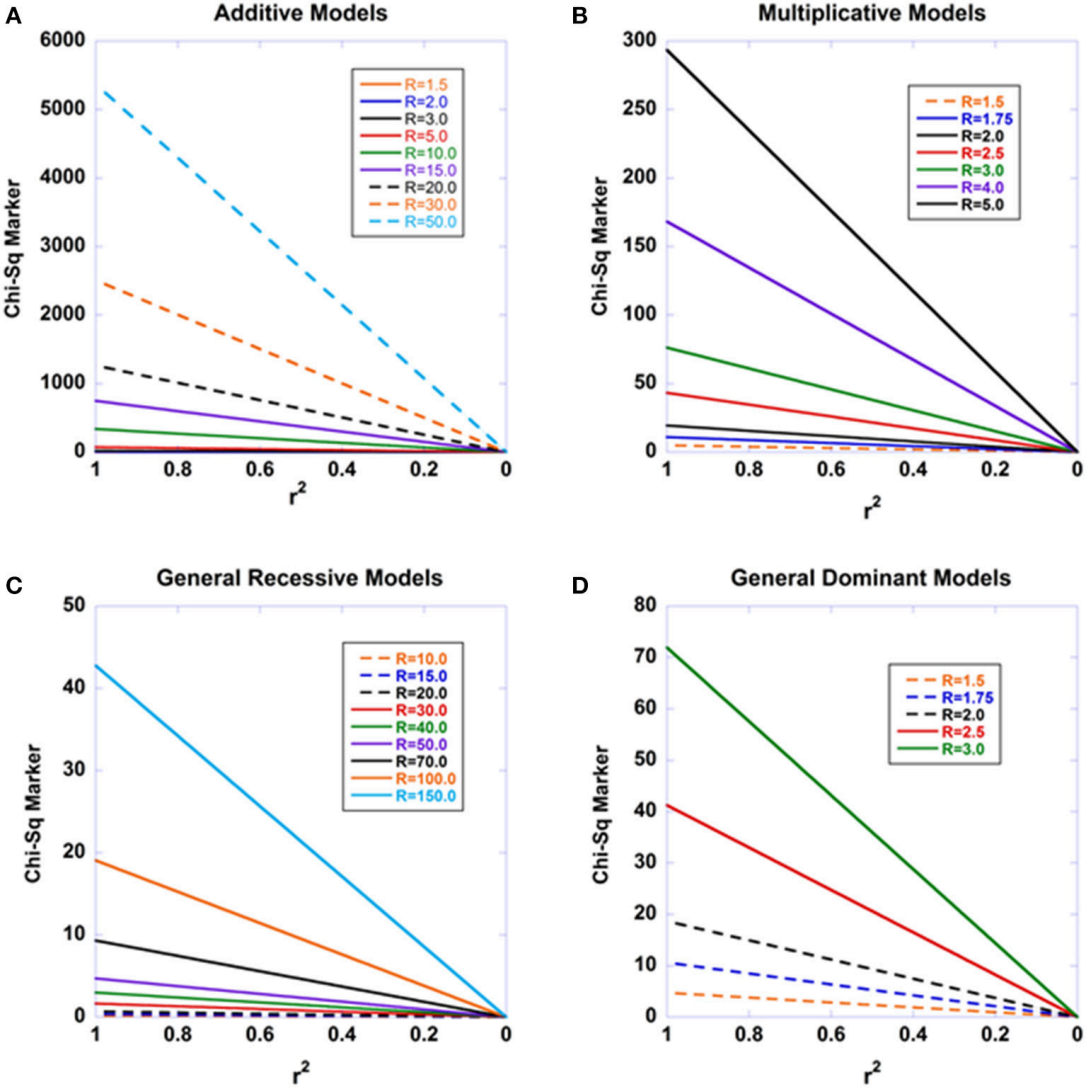
**The expected decay of disease association with declining linkage disequilibrium for four modes of inheritance**. The standard recursive haplotype frequencies under recombination were used to generate a series of haplotype combinations. The disease-predisposing allele at the causal site was set at a general population frequency of 0.01. The penetrance f_22_ was set to 0.001 and the remaining two penetrances varied according to the modes of inheritance examined and the relative risks (R) cited in the Figures. Sample sizes were set at *n*_D_ = 2000 and *n*_C_ = 2000. **(A)** displays the results for an additive model, such that f_12_ is the arithmetic mean of f_22_ and f_11_. **(B)** shows the results under a multiplicative model. **(C)** shows the results under a general recessive model. **(D)** shows the results under a general dominant model.

Plotting the Equation (2) approximation with the χ^2^ disease-association statistic on the ordinate and 1 − *r*^2^ on the abscissa is a simple method of displaying the expected linear decay in the χ^2^ -values as the linkage disequilibrium with a causal site declines at different marker sites. Figure 1 shows this relationship. This decay pattern was first used empirically in 2007–2008 to fine map the *TRAF1* region in rheumatoid arthritis (Schrodi et al., 2007a) and the *IL23R* region in psoriasis (Garcia et al., 2008) and has, in an analogous form, subsequently been used in other applications (Farh et al., 2015). Although this approximation is very useful in understanding the decay of disease association with declining linkage disequilibrium from a causal site, several simplifying assumptions were made in the original Pritchard-Przeworski derivation. While the impact of these assumptions have been explored to some extent in previous work (Hu et al., 2004), it is not known how violations of the original assumptions might produce departures from Equation (2) nor what the effect of sampling haplotypes does to the relationship. Hence, an exact relationship between disease association statistics and *r*^2^ -values with a causal site would aid in clarifying this relationship and motivate statistical approaches to harnessing this pattern for the purpose of fine-mapping functional alleles. Further, Monte Carlo simulations can be used to explore the how treating haplotype counts as random variables generates stochastic variation around this central relationship.

### Full derivation

In this section, we will show the algebraic relationship between the Chi-Square-test statistics at a causal site and marker site, without any assumptions regarding the probabilistic properties (or whether they are fixed parameters) of the allele frequencies or haplotype frequencies of which the statistics are composed. Note that in the Monte Carlo Simulations Section we will treat the haplotype counts as random variables; and hence the Chi-Squared statistics and *r*^2^ will each carry stochastic properties and we investigate these properties in that section.

Defining the Chi-Square-test statistics for a disease-causing site ($\chi_{D}^{2}$) and a marker closely linked to the disease site ($\chi_{M}^{2}$) following the Pritchard-Przeworski derivation,

(3) $$\begin{array}{l} \chi_{D}^{2}\;=\frac{{\left[p_{D}-p_{C}\right]}^{2}\left[2n\left(\frac{n_{D}}{n_{D}+n_{C}}\right)\left(\frac{n_{C}}{n_{D}+n_{C}}\right)\right]}{p\left(1-p\right)}, \end{array}$$

(4) $$\begin{array}{l} \chi_{M}^{2}\;=\frac{{\left[q_{D}-q_{C}\right]}^{2}\left[2n\left(\frac{n_{D}}{n_{D}+n_{C}}\right)\left(\frac{n_{C}}{n_{D}+n_{C}}\right)\right]}{q\left(1-q\right)}; \end{array}$$

where a two-site model is considered (site *A* segregating alleles *A*_1_ and *A*_2_, and site *B* segregating alleles *B*_1_ and *B*_2_), *p*, *p*_*D*_, and *p*_*C*_ are the frequencies of the *A*_1_ allele in the combined population, disease-affected population, and the control population, respectively, and where *q*, *q*_*D*_, and *q*_*C*_ are the frequencies of the *B*_1_ allele in the combined population, disease-affected population, and the control population, respectively. *n*_*D*_ and *n*_*C*_ are the sample sizes for diploid cases and controls, respectively, and *n* = *n*_*D*_ + *n*_*C*_. For this work, haplotype and allele probabilities conditional on disease status (i.e., within cases or within controls) are derived. For the haplotype and allele probabilities in the general population, we weighted the disease status conditional probabilities by the probability of disease or healthy control attributable to the causal site, in accordance with the law of total probability. Note, that the form of these Chi-Square statistics in Equations (3) and (4) is twice the value of traditionally-defined Chi-Square statistic. However, this scalar inflation factor cancels out in the subsequent derivation.

(5) $$\begin{array}{l} \frac{\chi_{M}^{2}\;}{\chi_{D}^{2}}=\frac{p\left(1-p\right){\left(q_{D}-q_{C}\right)}^{2}}{q\left(1-q\right){\left(p_{D}-p_{C}\right)}^{2}}. \end{array}$$

Noting that

$$\begin{array}{l} p=p_{D}K+p_{C}\left(1-K\right)\;and\;q=q_{D}K+q_{C}\left(1-K\right), \end{array}$$

where *K* is the *P*(*Case*) attributable to the causal site, we can substitute $p_{C}=\frac{p-Kp_{D}}{1-K}$ and $q_{C}=\frac{q-Kq_{D}}{1-K}$ into Equation (5), resulting in

(6) $$\begin{array}{l} \frac{\chi_{M}^{2}\;}{\chi_{D}^{2}}=\frac{p\left(1-p\right){\left(q-q_{D}\right)}^{2}}{q\left(1-q\right){\left(p-p_{D}\right)}^{2}}. \end{array}$$

This treatment of the allele frequencies using the law of total probability holds for all populations in which each individual is either a case or control (e.g., cohort studies or case/control study designs). The next aim in the derivation is to substitute quantities for the allele frequencies in the affected population at both sites in terms of penetrances, disease prevalence, and general population allele frequencies. The allele frequencies at both the causal and marker sites have been previously described for two-locus systems under general disease models (Schrodi et al., 2007b):

(7) $$\begin{array}{lll} p_{D} & = & \frac{p}{K}\left[f_{11}p+f_{12}\left(1-p\right)\right], \end{array}$$

(8) $$\begin{array}{lll} q_{D} & = & \frac{P_{11}}{K}\left[f_{11}p+f_{12}\left(1-p\right)\right]+\frac{P_{21}}{K}\left[f_{12}p+f_{22}\left(1-p\right)\right]; \end{array}$$

where *f*_11_, *f*_12_, *and f*_22_ are the prevalences of the *A*_1_*A*_1_, *A*_1_*A*_2_, *and A*_2_*A*_2_ genotypes, respectively, such that *f*_*ij*_ = *P*(*Case*|*A*_*i*_*A*_*j*_); which, under this monogenic model and assuming Hardy-Weinberg Equilibrium in the general population and using the law of total probability we can express the disease prevalence as, $K=f_{11}p^{2}\;+\;2f_{12}p\left(1-p\right)+\;f_{22}{\left(1-p\right)}^{2}$; and haplotype frequencies *P*_11_ = *P*(*A*_1_*B*_1_), and *P*_21_ = *P*(*A*_2_*B*_1_). Applied to complex diseases, it may be useful to think of this disease model as the subset of individuals with a common disease that is primarily driven by a particular locus. With the substitution into Equation (6),

(9) $$\begin{array}{l} \frac{\chi_{M}^{2}\;}{\chi_{D}^{2}}=\frac{p\left(1-p\right){\left\{\begin{array}{c} q-\frac{P_{11}}{K}\left[f_{11}p+f_{12}\left(1-p\right)\right]\\ -\frac{P_{21}}{K}\left[f_{12}p+f_{22}\left(1-p\right)\right] \end{array}\;\right\}}^{2}}{q\left(1-q\right){\left\{p-\frac{p}{K}\left[f_{11}p+f_{12}\left(1-p\right)\right]\right\}}^{2}}. \end{array}$$

In Equation (9), the R.H.S. numerator can be simplified to

$$\begin{array}{l} p\left(1-p\right)\left(\frac{1}{K^{2}}\right)\{P_{11}\left[f_{11}p+f_{12}\left(1-p\right)\right]\\ \;{+\;P_{21}\left[f_{12}p+f_{22}\left(1-p\right)\right]-Kq\}}^{2}. \end{array}$$

Noting that *P*_21_ = *q* − *P*_11_ and substituting $f_{11}p^{2}+2f_{12}p\left(1-p\right)+f_{22}{\left(1-p\right)}^{2}=K$, the numerator becomes

$$\begin{array}{l} p\left(1-p\right)\left(\frac{1}{K^{2}}\right){\left(P_{11}-pq\right)}^{2}{\left[f_{11}p+f_{12}\left(1-2p\right)-f_{22}\left(1-p\right)\right]}^{2}, \end{array}$$

whereas, the denominator in Equation (9) can be simplified to

$$\begin{array}{l} q\left(1-q\right)\left(\frac{1}{K^{2}}\right)p^{2}{\left[K-f_{11}p-f_{12}\left(1-p\right)\right]}^{2}. \end{array}$$

Hence, Equation (9) can be written as

(10) $$\begin{array}{l} \frac{\chi_{M}^{2}\;}{\chi_{D}^{2}}=\frac{D^{2}\left(1-p\right)}{pq\left(1-q\right)}\frac{{\left[f_{11}p+f_{12}\left(1-2p\right)-f_{22}\left(1-p\right)\right]}^{2}}{{\left[K-f_{11}p-f_{12}\left(1-p\right)\right]}^{2}}; \end{array}$$

where *D* = *P*_11_*P*_22_ − *P*_12_*P*_21_ = *P*_11_ − *pq*.

Again substituting $K=f_{11}p^{2}+2f_{12}p\left(1-p\right)+f_{22}{\left(1-p\right)}^{2}$,

(11) $$\begin{array}{lll} \frac{\chi_{M}^{2}\;}{\chi_{D}^{2}} & = & \frac{D^{2}\left(1-p\right)}{pq\left(1-q\right)}\frac{{\left[f_{11}p+f_{12}\left(1-2p\right)-f_{22}\left(1-p\right)\right]}^{2}}{{\left[\left(1-p\right)\left(-f_{11}p-f_{12}\left(1-2p\right)+f_{22}\left(1-p\right)\right)\right]}^{2}}\\ = & \frac{D^{2}}{pq\left(1-p\right)\left(1-q\right)}{\left[\frac{f_{11}p+f_{12}\left(1-2p\right)-f_{22}\left(1-p\right)}{f_{11}p+f_{12}\left(1-2p\right)-f_{22}\left(1-p\right)}\right]}^{2} \end{array}$$

(12) $$\begin{array}{ll} = & \frac{D^{2}}{pq\left(1-p\right)\left(1-q\right)}\\ = & r^{2}. \end{array}$$

Therefore, we have shown the exact relationship under our model,

(13) $$\begin{array}{l} \chi_{M}^{2}=r^{2}\chi_{D}^{2}. \end{array}$$

**Figure 2:**
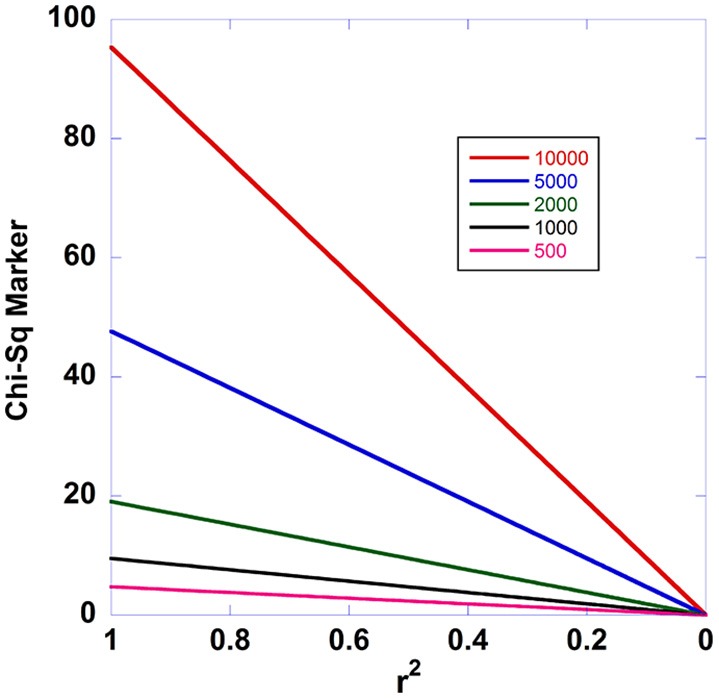
**Effect of sample size on the expected decay of disease association with declining linkage disequilibrium**. This figure shows how the fine mapping theorem behaves under different sample sizes. The case/control sample sizes in the two-site model are varied from 500 to 10,000.

Not only is this relationship an exact result under the model employed, but it is universal in that there is no dependence on the penetrances. Thus, we may expect that from a true disease-susceptibility site, that there should be a linear decay in the Chi-square statistics for disease association with declining *r*^2^ -values with the causal site. Figure 1 shows the expected disease association decay with declining linkage disequilibrium from the causal site for additive, multiplicative, recessive, and dominant sets of models. The patterns arising from various relative risks are presented. Similarly, Figure 2 presents the patterns expected as a function of sample sizes. Aside from Equation (13) illuminating a central aspect of disease genetics, we suspect that it carries utility in fine mapping applications—we hypothesize that identifying this type of pattern in fine mapping data will better enable the pinpointing of truly causal sites through harnessing correlated data.

### Corollary

Consider the situation where there is a disease-susceptibility site and other sites in differing levels of linkage disequilibrium with the disease-susceptibility site. From large-scale genotyping or sequencing studies, we often know the matrix of pairwise *r*^2^ -values, and allele frequencies at each site in the general population, broadly defined. An interesting question arises: If one has genotyped a marker site in a case/control sample set and calculated $\chi_{M}^{2}$ testing for disease association, can we infer the expected effect size at a non-interrogated causal site? Using Equation (13), and substituting allele frequencies at the causal site,

(14) $$\begin{array}{l} \frac{\chi_{M}^{2}}{r^{2}}=\frac{n_{e}{\left(p_{D}-p_{C}\right)}^{2}}{2p\left(1-p\right)}; \end{array}$$

where $n_{e}=\frac{4n_{D}n_{C}}{n_{D}+n_{C}}$, the effective total number of independent diploid samples. Defining a traditional allelic odds ratio, *R*, calculated at the causal site as

$$\begin{array}{l} R=p_{D}\left(1-p_{C}\right){\left[p_{C}\left(1-p_{D}\right)\right]}^{-1}, \end{array}$$

the allele frequency in the cases can be solved: $p_{D}=\frac{Rp_{C}}{1-p_{C}+Rp_{C}}$.

Therefore,

(15) $$\begin{array}{l} {\left(\frac{Rp_{C}}{1-p_{C}+Rp_{C}}-p_{C}\;\right)}^{2}=2p\left(1-p\right)\frac{\chi_{M}^{2}}{n_{e}r^{2}}. \end{array}$$

To simplify the derivation, we will assume that the disease studied is not very common such that the allele frequency in controls is well-approximated by the allele frequency in the general population, *p*_*C*_ ≅ *p*. This is also true if samples drawn from the general population are serving as the controls. Hence,

(16) $$\begin{array}{l} \frac{Rp}{1-p+Rp}=p+\left(\frac{Z_{M}}{r}\right)\sqrt{\frac{2p\left(1-p\right)}{n_{e}}}. \end{array}$$

Solving for *R*,

(17) $$\begin{array}{l} R=\;\left(\frac{1-p}{p}\right)\;[\frac{p+\sqrt{\frac{2p\left(1-p\right)\chi_{M}^{2}}{n_{e}r^{2}}}}{1-p-\sqrt{\frac{2p\left(1-p\right)\chi_{M}^{2}}{n_{e}r^{2}}}}]. \end{array}$$

**Figure 3:**
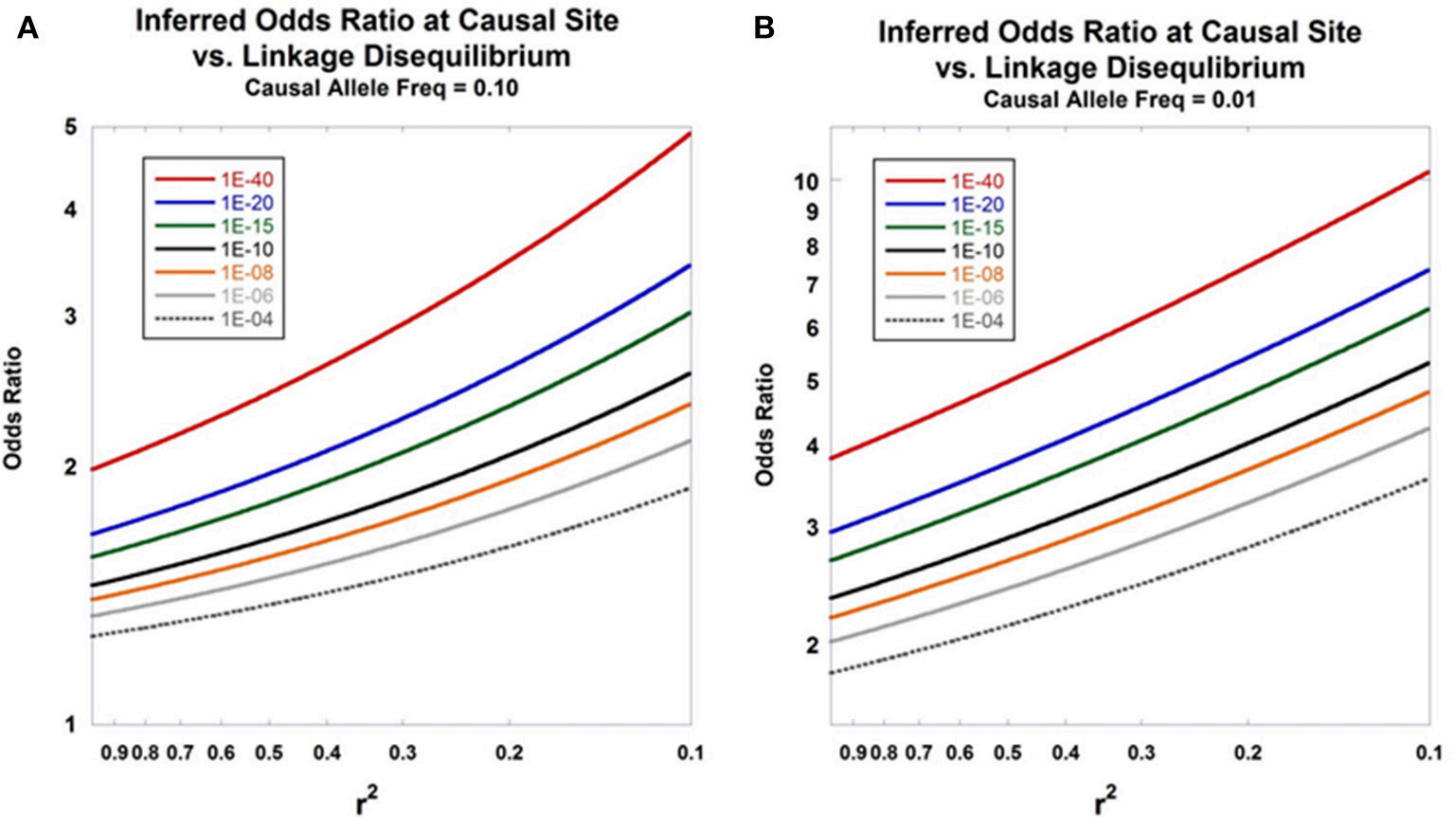
**Inferred odds ratio**. The relationship between the inferred odds ratio at a causal site and the level of linkage disequilibrium with an interrogated marker is presented in **(A,B)**. Equation (17) is used for the calculations. The seven curves show the patterns of expected odds ratios for disease association at the causal site under different observed *p*-values calculated at the marker site. Sample size was set at *n*_e_ = 5000. **(A)** shows results assuming that the disease-predisposing allele at the causal site has frequency of 0.10 in the general population, whereas **(B)** sets that frequency at 0.01.

To illustrate the use and implications of Equation (17), suppose that we have genotyped a site in 500 diploid cases and 500 diploid controls and calculated the test statistic χ^2^ = 20, corresponding to *p* = 1.57E-03 (recall that half the Pritchard-Przeworski statistic is Chi-Square distributed with one degree of freedom). Further assume that this region has previously been subjected to next-generation sequencing in individuals derived from the same source population as the cases and controls which has yielded the discovery of numerous additional variants closely linked to the genotyped site, allele frequencies at those variants, and an array of pairwise linkage disequilibrium values across the region of interest. Under that scenario, one would typically have access to good estimates of the general population allele frequencies and *r*^2^ -values at sites neighboring the genotyped site that produced the original finding. Suppose that one of these adjacent sites has a general population allele frequency *p* = 0.03 and a linkage disequilibrium value with the genotyped site of *r*^2^ = 0.2. Under the two-site model, we would therefore estimate the odds ratio at the putative, non-genotyped, causal site to be 5.17. Put another way, the putative causal site, having the general population allele frequency and linkage disequilibrium values above, would have to have an odds ratio of 5.17 in order to generate twice a standard Chi-Square statistic value at the genotyped site of 20 given 500 cases and 500 controls. Indirect inference of the properties of non-interrogated causal sites can be helpful in subsequent experimental efforts to identify disease-predisposing sites in a fine-mapped region. Figure 3 displays the relationship between the inferred odds ratio at the causal site from disease association data at the marker site as a function of linkage disequilibrium between the two sites. Graphs for various *p*-values at marker site are shown. Additional work under a stochastic model would enable the calculation of the posterior probabilities of properties of non-interrogated causal sites given genetic data at linked markers.

The results detailed in Equations (1–17) do not treat any of the parameters, such as haplotype frequencies, as random variables. Clearly, haplotype counts in cases and controls should be treated with sampling processes from a larger population. To address this issue, we have constructed a Monte Carlo simulation program to generate haplotypes under a probabilistic model. Under this program we are able to explore the variation around Equation (13) generated by sampling haplotypes and to observe effects that may be produced by different sets of parameters.

### Monte carlo simulations

In an effort to understand the variation in the patterns of disease association decay as a function of linkage disequilibrium with a causative site, we constructed a Monte Carlo simulation using a generalized disease model (penetrances for each of the three genotypes at the causal site are parameterized) and treating the haplotype counts in cases and controls as random variables. Recombination was introduced between a causal site and a closely linked marker as a realistic method of generating different sets of 2-site haplotypes for the general population (Hartl and Clark, 1989). For a rate of recombination, *c*, and generation time *t*, we used the following set of recursions (Haldane model of recombination):

(18) $$\begin{array}{lll} P_{11,t} & = & P_{11,t-1}\left(1-c\right)+cpq, \end{array}$$

(19) $$\begin{array}{lll} P_{12,t} & = & P_{12,t-1}\left(1-c\right)+cp\left(1-q\right), \end{array}$$

(20) $$\begin{array}{lll} P_{21,t} & = & P_{21,t-1}\left(1-c\right)+c\left(1-p\right)q, \end{array}$$

(21) $$\begin{array}{lll} P_{22,t} & = & P_{22,t-1}\left(1-c\right)+c\left(1-p\right)\left(1-q\right). \end{array}$$

Hence, for the general population, we can express *r*^2^ as a function of generation time using the recursions in Equations (18–21):

(22) $$\begin{array}{l} r_{t}^{2}=\frac{{\left(1-c\right)}^{2}{\left(P_{11,t-1}P_{22,t-1}-P_{12,t-1}P_{21,t-1}\right)}^{2}}{\begin{array}{c} \left(P_{11,t-1}+P_{12,t-1}\right)\left(P_{21,t-1}+P_{22,t-1}\right)\\ \left(P_{12,t-1}+P_{22,t-1}\right)\left(P_{11,t-1}+P_{21,t-1}\right) \end{array}}. \end{array}$$

Assuming Hardy-Weinberg equilibrium in the general population at both sites, the proportion of individuals affected by the disease attributable to this locus, is calculated through the previously-described formula for disease prevalence. To calculate the expected haplotype frequencies in cases, we used Bayes theorem. Hence, the expected frequency of the *A*_1_*B*_1_ haplotype in cases is

(23) $$\begin{array}{l} V_{11}=\frac{P_{11}}{K}\left[f_{11}p_{1}+f_{12}\left(1-p_{1}\right)\right]. \end{array}$$

In an analogous manner, the remaining haplotype frequencies in cases, where the subscript indicates the haplotype, are

(24) $$\begin{array}{lll} V_{12} & = & \frac{P_{12}}{K}\left[f_{11}p_{1}+f_{12}\left(1-p_{1}\right)\right], \end{array}$$

(25) $$\begin{array}{lll} V_{21} & = & \frac{P_{21}}{K}\left[f_{12}p_{1}+f_{22}\left(1-p_{1}\right)\right], \end{array}$$

(26) $$\begin{array}{lll} V_{22} & = & \frac{P_{22}}{K}\left[f_{12}p_{1}+f_{22}\left(1-p_{1}\right)\right]. \end{array}$$

The haplotype frequencies in controls are simply

(27) $$\begin{array}{lll} U_{11} & = & \frac{\left(P_{11}-V_{11}K\right)}{1-K}, \end{array}$$

(28) $$\begin{array}{lll} U_{12} & = & \frac{\left(P_{12}-V_{12}K\right)}{1-K}, \end{array}$$

(29) $$\begin{array}{lll} U_{21} & = & \frac{\left(P_{21}-V_{21}K\right)}{1-K}, \end{array}$$

(30) $$\begin{array}{lll} U_{22} & = & \frac{\left(P_{22}-V_{22}K\right)}{1-K}. \end{array}$$

Sampling of the case and control haplotypes from the expected frequencies is accomplished through two independent multinomial variates such that the joint densities are given by

(31) $$\begin{array}{l} P\left(X_{11}=x_{11},X_{12}=x_{12},X_{21}=x_{21},X_{22}=x_{22}\right)\\ =n_{D}!\left(\frac{V_{11,t}^{x_{11}}V_{12,t}^{x_{12}}V_{21,t}^{x_{21}}V_{22,t}^{x_{22}}}{x_{11}!x_{12}!x_{21}!x_{22}!}\right), \end{array}$$

(32) $$\begin{array}{l} P\left(Y_{11}=y_{11},Y_{12}=y_{12},Y_{21}=y_{21},Y_{22}=y_{22}\right)\\ =n_{C}!\left(\frac{U_{11,t}^{y_{11}}U_{12,t}^{y_{12}}U_{21,t}^{y_{21}}U_{22,t}^{y_{22}}}{y_{11}!y_{12}!y_{21}!y_{22}!}\right)\;. \end{array}$$

Hence, the sample frequency of the causal allele in cases and controls, respectively, are

(33) $$\begin{array}{l} {\hat{p}}_{D}={\left(n_{D}\right)}^{-1}\left(x_{11}+x_{12}\right), \end{array}$$

and

(34) $$\begin{array}{l} {\hat{p}}_{C}={\left(n_{C}\right)}^{-1}\left(y_{11}+y_{12}\right). \end{array}$$

**Figure 4:**
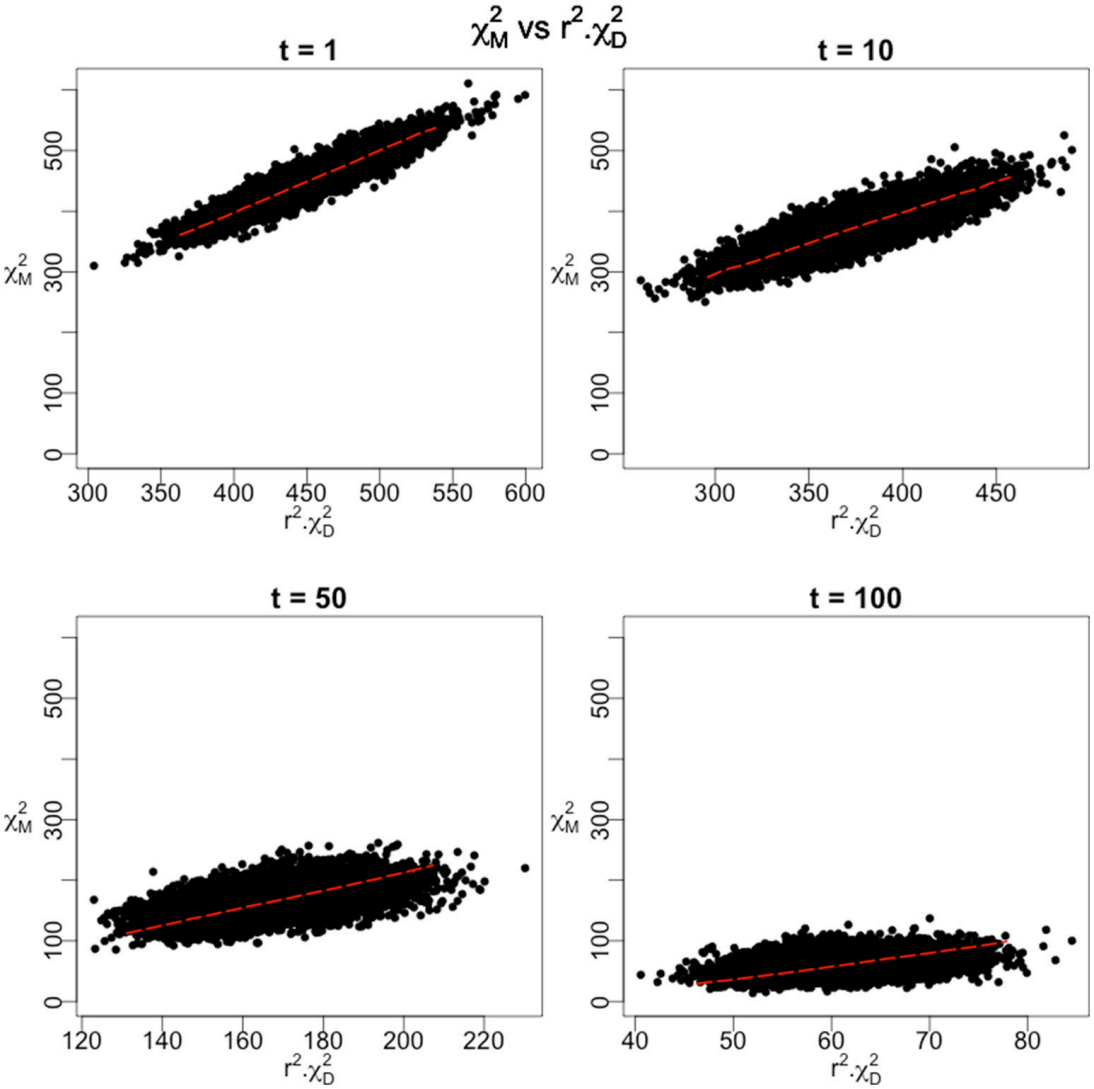
**Monte Carlo results under the 2-site model with recombination**. The Chi-Square statistic as measured at the marker site is plotted against the product of *r*^2^ and the Chi-Square Statistic at the disease site for 10,000 replications of the simulation. 10,000 disease cases and 40,000 controls were assumed in the calculations. The initial frequencies of the four haplotypes were 0.70 for the parental, non-causal haplotype, 0.28 for the parental haplotype carrying the causal variant, and 0.01 for each of the recombinant haplotypes. As time (t) increases, these frequencies varied according to the recursions specified in Equations (18–21). An additive model was assumed as the mode of inheritance model with penetrances of 0.01, 0.03, and 0.05 for the three genotypes at the causal site.

**Figure 5:**
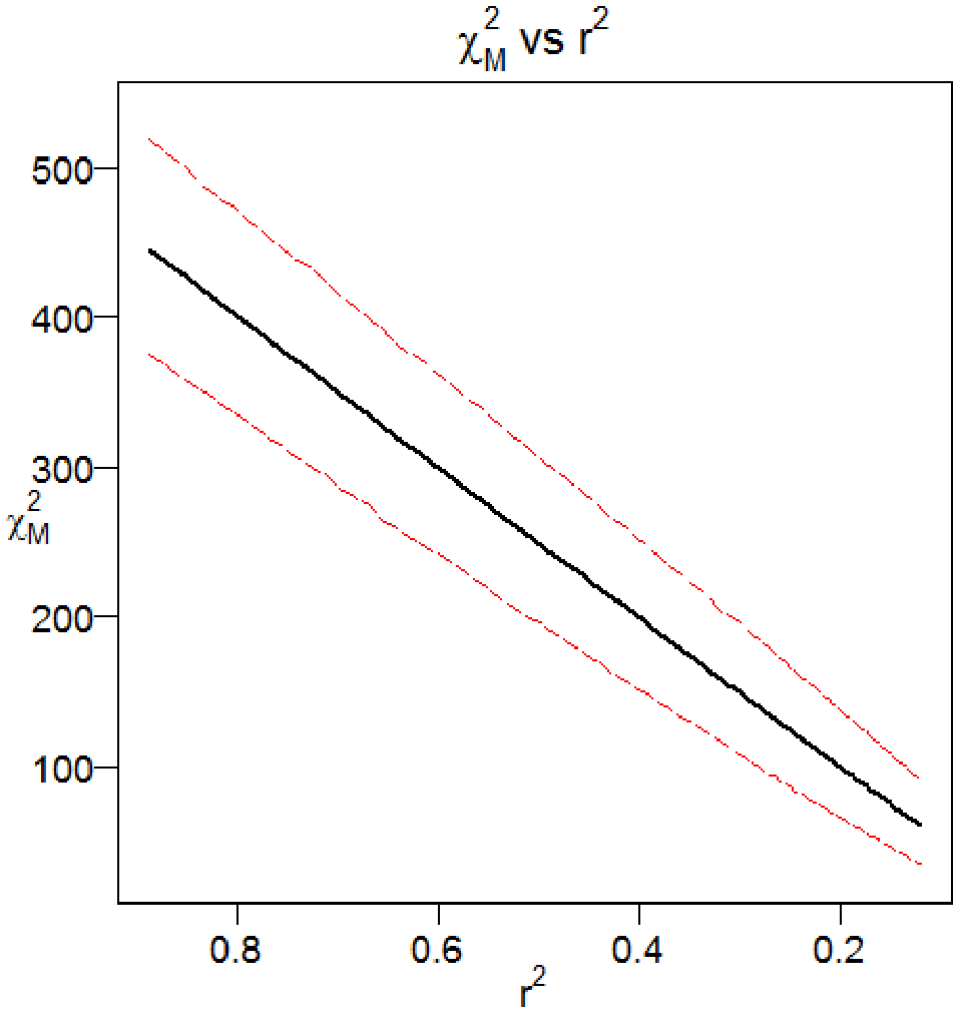
**Mean and 95% Confidence Interval for the Chi-Square Statistic at the Marker Site as a Function of Linkage Disequilibrium**. Results from the Monte Carlo simulation showing the mean and 95% confidence interval for the Chi-Square statistic at the marker site, plotted as a function of *r*^2^ with the causal site. The two-site model with recombination and additive mode of inheritance described previously were used.

We employed an additive model for the penetrances at the causal site and a design using 10,000 cases and 40,000 controls. As the time parameter is increased, the number of recombination events between the casual site and the marker site increases and there is a corresponding reduction in the linkage disequilibrium between the two sites. Figure 4 shows the distribution of the association statistic at the marker site (Equation 4) plotted against the product of the association statistic at the causal site (Equation 3) and the $r_{t}^{2}$ -value between the two sites. Four different time points were evaluated in the simulation, each with 10,000 replicates generated. The patterns show the general linear trend of how the association statistics scale with linkage disequilibrium and the variation around this pattern. For fixed properties at a causal site, Figure 5 displays the mean value and 95% confidence interval of the association statistic at the marker site as the $r_{t}^{2}$ -value declines.

### Application to experimental data

**Figure 6:**
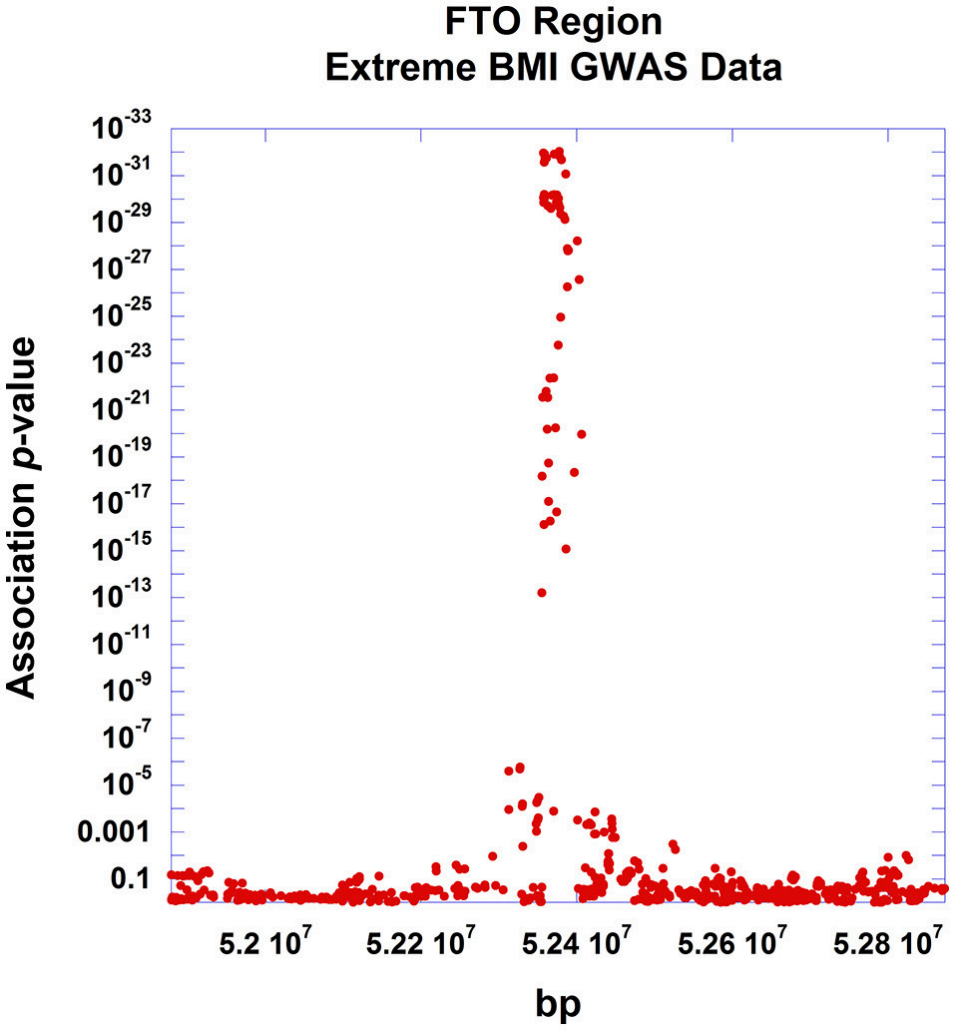
**Positional association plot of SNPs around the *FTO* gene region**. Association data from the Berndt et al. study of extreme BMI were used for this positional plot. SNPs (*n* = 752) within 500 kb of the *FTO* gene, having linkage disequilibrium information from the 1000 genomes study were used in the plot showing strong localization of the association signal around the *FTO* gene.

**Figure 7:**
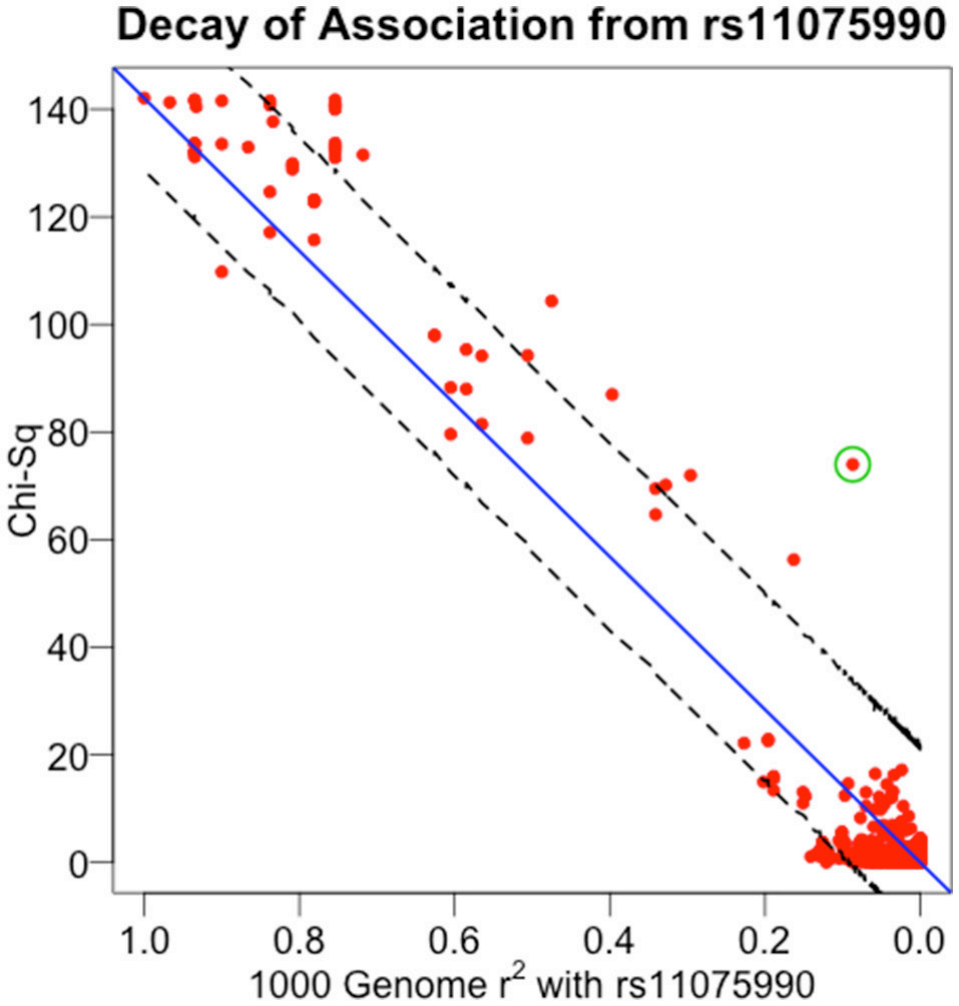
**Decay of association from rs11075990**. Association from the Berndt et al. study of extreme BMI was plotted against the pairwise linkage disequilibrium (*r*^2^) values of each SNP and rs11075990—the most significant finding in the region. rs2058908 is denoted with the green circle. Ninety-five percent of confidence intervals are determined through the resampling scheme presented in the text.

All indicated earlier, there are several uses of the theorem presented here. The pattern of linear decay of association (as measured by the test statistic) with declining linkage disequilibrium can be used to support various markers as causal sites. Conversely, significant departure from the expected pattern can indicate multiple causal sites segregating at the disease locus. And additionally, understanding this fine mapping theorem can be used to infer properties of non-interrogated causal sites. To illustrate the application of the relationship described in Equation (13) to experimental data, we used GWAS data around the well-established obesity locus, *FTO*, generated by a recent large study of extreme BMI (Berndt et al., 2013). The *FTO* gene encodes for an alpha-ketoglutarate-dependent dioxygenase (Gerken et al., 2007), playing a role in growth and development (Boissel et al., 2009; Daoud et al., 2016), and has been reliably associated with the related conditions of type 2 diabetes, BMI, adiposity and other obesity-related traits (Scott et al., 2007; Zeggini et al., 2007; Lindgren et al., 2009; Thorleifsson et al., 2009; Fox et al., 2012; DIAGRAM Consortium et al., 2014; Wood et al., 2016). Within the *FTO* gene region, the study found that rs11075990 exhibited the strongest association with extreme BMI with a reported *p*-value of 9.3E-33. From this study, we identified 752 SNPs residing within a ~1 Mb region surrounding *FTO*, having linkage disequilibrium data from the 1000 Genomes project (The 1000 Genomes Project Consortium et al., 2015). Figure 6 displays the positional association of these data, showing a substantial peak localized on chr16q over the *FTO* gene. Plotting these association results as a function of pairwise linkage disequilibrium (as measured by *r*^2^) with rs11075990, there is a general decay of the Chi-Square association statistics with declining *r*^2^ -values (Figure 7). Pearson's correlation is 0.979 and the *p*-value for this relationship (testing Spearman's rho under the null model of no correlation) is 2.87E-29. For this example, there are some immediate findings by visual inspection. The general pattern following the theorem is present. In addition, there appear to be some SNPs with extreme BMI associations that substantially exceed the level of association expected to be driven through linkage disequilibrium with rs11075990. That is, the theoretical model of one causal site (rs11075990) driving the extreme BMI association patterns in the *FTO* gene region may not explain the association statistics at some SNPs, such as rs2058908, where the theory only predicts a Chi-Square-value of 12.36 (*r*^2^ with rs11075990 is 0.087) and yet the observed Chi-Square statistic is 73.98. Hence, the genetic information at rs2058908 may be driven by a causal signal independent of rs11075990 (rs2058908 is denoted with a green circle in Figure 7). A test of conditional association could be used to verify these types of hypotheses. Since the residuals obtains from the fitted line (the line that passes through the origin and the Chi-Square value associated to the causal site) and the observed Chi-Square-values are not normally-distributed, we used a resampling approach to obtain a 95% confidence band (dashed lines in Figure 7). In this approach, we treat the fitted Chi-Square-values to be the expected response for the bootstrap samples, and by resampling the original residuals, we obtain bootstrap replicates for the fixed covariate (*r*^2^) (Fox and Weisberg, 2012). Here, we resampled the original residuals 100,000 times in the R programming language (R Core Team, 2014) and used the 0.025 and 0.975 quantiles of the resampled fits to achieve the 95% confidence band in Figure 7.

## Discussion

One of the most fundamental patterns in disease genetics is the nature of the decay of disease association with declining linkage disequilibrium from a causal site. Motivated by the Kruglyak and Pritchard-Przeworski derivations for the approximate increase in sample size to attain the equivalent statistical power at a marker site in linkage disequilibrium with a causal site, we first showed how this result could be used to produce an approximation showing a linear relationship in the Chi-Square association statistics testing disease association at a marker and a causal site and that the ratio of the two was approximately *r*^2^ (Equation 2). Next, using a general two-site model with penetrances, we showed that this is indeed an exact result and invariant to the mode of inheritance model (Equation 13). In this derivation, we treated the variables as fixed parameters. To treat the situation where the haplotype frequencies have sampling properties (i.e., are treated as random variables), we wrote a Monte Carlo simulation of this system for finite sample sizes and used a standard model of recombination between the causal and marker sites. The results characterized the stochastic variability around the initial result. Lastly, we applied this work to experimental data from a large GWAS on extreme BMI and showed reasonably good correspondence with this fine mapping theory.

Aside from being a theorem in disease genetics for dichotomous traits, we hope that this fine mapping theorem can serve as an aid in identifying casual variants segregating in a region associated with disease. Recently, substantial effort has driven the field of fine-mapping forward. To address the statistical aspects of prioritizing potentially causal variants within a fine-mapped region, several methods have been developed including a useful Bayesian method created by Maller et al. (The Wellcome Trust Case Control Consortium et al., 2012), which uses Bayes Factor for each variant in the region and calculates the proportion of the total sum of Bayes Factors in the region that is attributable to that variant, producing a relative ranking of the strength of evidence for each variant within the disease-associated region being causal. These calculations allow for the determination of a credible set of highest ranked variants that explains the large majority of the statistical evidence of disease association within the region of interest. The Maller et al. method has been applied to fine mapping data for complex diseases, such as type 1 diabetes (Onengut-Gumuscu et al., 2015). Other important developments in fine mapping approaches include: Bim-Bam (Servin and Stephens, 2007), another Bayesian approach which determines subsets of variants that likely contain causal sites, CAVIAR (Hormozdiari et al., 2014) and CAVIARBF (Chen et al., 2015), coalescent-based methods (Graham, 1998; Morris et al., 2002; Zöllner and Pritchard, 2005), and PAINTOR (Kichaev et al., 2014), which incorporates functional annotation data in a probabilistic manner. Several different extensions of the work presented here could substantially aid fine mapping efforts for complex diseases: (1) Statistical approaches that harness the pattern of association decay with declining linkage disequilibrium will leverage the genetic data at a fine-mapped region to better support or reject the hypothesis that a particular site is indeed causal. Screening each site for a goodness-of-fit with the expected decay pattern from a causal site would better enable the detection of causal sites; (2) Future work focusing on imputing additional properties of a non-interrogated causal variant within a disease-associated region using the linkage disequilibrium patterns and disease association statistics would provide valuable insights into design and interpretation of fine mapping studies. For example, if one imputed a low-frequency, high effect size variant, then experimental designs and genetic techniques, such as sequencing, that have high power to detect such variants can be utilized; and (3) It is becoming increasingly clear that the large majority of regions associated with complex disease susceptibility have multiple predisposing alleles segregating in the populations examined. Methods that extend the simple two-site model explored here to include multiple causal sites will be invaluable for the identification of these functional variants.

## Author contributions

MM made substantial contributions to the analysis and interpretation of data, constructed the Monte Carlo simulations, devised the method for obtaining confidence intervals and generated figures and drafting/revising the manuscript. NB made substantial contributions to the interpretation of data, proofing the derivations, and editing the manuscript. JU, MF, XL, and ZY reviewed the manuscript, proofed the derivations, and aided in analyses. MH and SH reviewed and edited the manuscript. SS originated the concept of the manuscript, designed the study, derived the equations, aided in the design of the simulations, interpreted the data, generated figures and drafted/revised the manuscript.

### Conflict of interest statement

The authors declare that the research was conducted in the absence of any commercial or financial relationships that could be construed as a potential conflict of interest.
